# Influence of structural colour on the photoprotective mechanism in the gametophyte phase of the red alga *Chondrus crispus*

**DOI:** 10.1098/rsif.2023.0676

**Published:** 2024-02-21

**Authors:** Ariel García Fleitas, Samim Sardar, Margot Minju Arnould-Pétré, Maria Murace, Silvia Vignolini, Juliet Brodie, Guglielmo Lanzani, Cosimo D'Andrea

**Affiliations:** ^1^ Center for Nano Science and Technology, Istituto Italiano di Tecnologia, Via Rubattino 81, 20134 Milano, Italy; ^2^ Dipartimento di Fisica, Politecnico di Milano, Piazza L. da Vinci 32, 20133 Milano, Italy; ^3^ Natural History Museum, Science, Cromwell Road, London SW7 5BD, UK; ^4^ Department of Chemistry, University of Cambridge, Lensfield Road, Cambridge CB2 1EW, UK

**Keywords:** energy transfer, molecular mechanism, photoprotection, phycobilisomes, time-resolved fluorescence spectroscopy

## Abstract

Marine life is populated by a huge diversity of organisms with an incredible range of colour. While structural colour mechanisms and functions are usually well studied in marine animal species, there is a huge knowledge gap regarding the marine macroalgae (red, green and brown seaweeds) that have structural coloration and the biological significance of this phenomenon in these photosynthetic organisms. Here we show that structural colour in the gametophyte life history phase of the red alga *Chondrus crispus* plays an important role as a photoprotective mechanism in synergy with the other pigments present. In particular, we have demonstrated that blue structural coloration attenuates the more energetic light while simultaneously favouring green and red light harvesting through the external antennae (phycobilisomes) which possess an intensity-dependent photoprotection mechanism. These insights into the relationship between structural colour and photosynthetic light management further our understanding of the mechanisms involved.

## 1. Introduction

The brightest colours in nature are often obtained by the interaction of light with ordered nanostructured materials (often referred to as photonic crystals) through interference [1,2]. These structures are widespread in terrestrial [3,4] and marine animals [5] and their role is well understood in insects in terms of communication, mate attraction and predation, impacting the individual's chances of reproducing and surviving [6,7]. However, structural colours are also present in photosynthetic organisms including red, green and brown macroalgae [8], diatoms [9], and land plants [10]. In the macroalgae, the biological function of structural colour remains unclear. It has been hypothesized that structural colour may have a photoprotective function in these organisms [8,11] and/or provide a mechanism that produces an increase in photosynthesis [12] but the impact of structural colours on the molecular photosynthetic machinery is still unknown [8,13].

*Chondrus crispus* (Irish moss) is a common red alga on rocky shores and in the shallow subtidal in the North Atlantic and an example of a seaweed that exhibits structural colour, which is perceived by eye as blue iridescence on the tips of its fronds. As a traditional source of carrageenan, the valuable polysaccharide used in the food and other industries, its biology has been extremely well studied over many years and it was one of the first red algae to have its whole genome sequenced [14]. It has a triphasic life history [15] consisting of (i) the dioecious haploid female and male gametophytes, (ii) the diploid carposporophyte which develops directly on the female gametophyte, and (iii) the tetrasporophyte which is morphologically similar in appearance to the gametophyte phase [15].

Structural colour in *C. crispus* only occurs in the gametophyte stage of the life history and is localized at approximately 1.5 cm from the tip of the frond [16]. Chandler *et al.* [11] demonstrated that the blue structural colour on the surface of the fronds was produced as a result of the dimensions and organization of several cuticular layers (lamellae) and suggested that its presence in the alga was likely to be influenced by local factors such as radiation intensity and turbidity of the water [8], suggesting a photoprotective function [11].

Red algae, as oxygenic photosynthetic organisms, convert sunlight into chemical energy and contain two photosystems, photosystem I (PSI) and photosystem II (PSII), located within the thylakoid membrane of the chloroplast [17]. Both PSI and PSII contain their own reaction centres inside these protein complexes. PSII functions primarily in the initial light-harvesting and water-splitting reactions, while PSI is responsible for electron transfer and ultimately NADPH production in the later stages of photosynthesis [18,19]. As the light sensitivity of the reaction centre is limited to the blue and red spectral region (*ca* 440 and 680 nm), some photosynthetic organisms assemble unique antenna pigment–protein complexes to absorb photons from other spectral regions and efficiently transfer this energy to the reaction centre [20]. Red algae contain PSI which includes light-harvesting complex I (LHCI) antenna and PSII which contains an external antenna system, the phycobilisome which is attached to the stromal side of thylakoid membranes of the chloroplast in a highly aggregated form [21]. Phycobilisomes contain chromophore-linked phycobiliproteins and colourless hydrophobic linker polypeptides. Among the phycobiliproteins, allophycocyanin is at the core of the phycobilisomes, surrounded by rods containing only phycocyanin or a combination of phycoerythrin and phycocyanin, depending on the species [22,23]. Therefore, an energy transfer chain is formed in the thylakoid membrane following the pathway: phycoerythrin → phycocyanin → allophycocyanin → chlorophyll [24,25]. Linker polypeptides support the assembly of the proteins which indirectly affect the energy transfer chain through its orientation and directly because some of them are also biliproteins working as intermediary acceptors and donors [26].

In order to achieve maximum efficiency during oxygenic photosynthesis, a balanced distribution of absorbed light energy between PSI and PSII needs to be maintained [27]. Moreover, under high light conditions, the maximum electron transport rate is eventually exceeded, and undesired photochemistry occurs, leading to the production of reactive oxygen species which can damage the photosynthetic apparatus. Therefore, regulatory mechanisms called photoprotection mechanisms have evolved to cope with variations in light intensity and ensure the smooth functioning of the photosynthetic machinery. However, photoprotection mechanisms in red algae have not been intensively studied at the level of the external antenna [28]. Two important light management mechanisms that act in the external antennae of red algae are: (i) energy spill over between both photosystems where phycobilisomes transfer energy not only to PSII but also to PSI [29,30] and (ii) state transitions, involving phycobilisome mobility [30–33]. *Chondrus crispus* presents a spill over quenching mechanism through which the amount of light received for each photosystem is regulated [32]. Moreover, Lu-Ning Liu *et al.* [34], working on the unicellular red algae *Porphyridium cruentum*, proposed another photoprotection mechanism in phycobilisomes involving a light-induced phycoerythrin decoupling as a strategy to block the transfer from phycoerythrin to phycocyanin*.* Yu-Hao Chiang *et al.* [35] suggested that the decoupling of the phycobilisome of photosystem II was the dominant process involved in non-photochemical quenching through conformational changes in the extremophilic red alga *Cyanidioschyzon merolae*.

The study of the photo-physics of energy transfer processes in photosynthetic organisms has been made possible using time-resolved optical spectroscopy (time-resolved fluorescence spectroscopy and transient absorption) [36,37] and been able to confirm the events that occur in sequential energy transfer processes in extracted biliproteins [22,23,29,36–43].

The red alga *C. crispus*, because it only has structural colour in one life history phase (gametophyte), makes an excellent model with which to study light management in the presence and absence of structural colour. Here, we report on the influence of structural coloration on the molecular mechanism of photosynthesis by comparing intact *C. crispus* gametophytes and tetrasporophytes using time-resolved fluorescence spectroscopy.

## 2. Results

**Figure 1.**
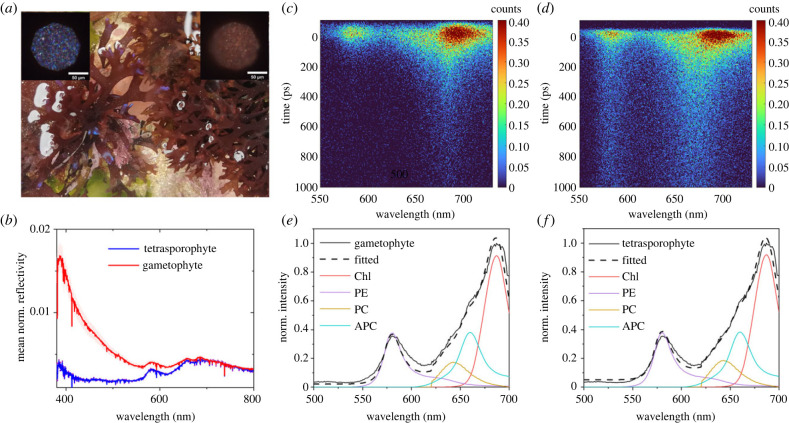
Characterization of *Chondrus crispus*: (*a*) gametophyte, with structural colour at the frond tips (left); tetrasporophyte, without structural colour (right). Insets show corresponding microscopic images of the gametophyte (left) and tetrasporophyte (right) tips. (*b*) Reflectivity spectra of gametophytes (35 replicas from 11 samples, multiple tips were used for measurements from a single sample) and tetrasporophytes (35 replicas from seven samples) with the associated standard deviation shown as a pink and purple area, respectively. Time-resolved fluorescence maps of (*c*) gametophyte and (*d*) tetrasporophyte tips upon excitation at 400 nm and (*e,f*) spectra integrated over a 2 ns window and spectra of the individual pigments of the algae [44] assigned to chlorophyll (Chl) [45,46], phycoerythrin (PE), phycocyanin (PC), and allophycocyanin (APC) [43,47] and the fitted spectrum using the spectra of the individual pigments. The violet, orange, blue and red lines are the spectra of PE, PC, APC and Chl with the amplitudes obtained by fitting to the experimental data and the dashed line is the spectrum generated by the superposition of these spectra.

The presence of structural colour as intensely blue coloured frond tips in the gametophyte and its absence in the tetrasporophyte phase *in situ Chondrus crispus* (figure 1*a*) was confirmed by the reflectivity spectra of the two life history phases (figure 1*b*). Reflectivity spectra of both gametophyte and tetrasporophyte showed a peak near 400 nm, which was higher in the gametophyte due to the multi-layered structure. By averaging over 35 reflectivity spectra, the mean reflectivity at 400 nm was 0.014 for gametophyte samples, and 0.0025 for tetrasporophyte samples, respectively.

As the maximum reflectivity of the photonic multi-layer was near 400 nm, we selected that wavelength to excite the samples to follow the energy transfer pathways in the antennae of the alga with time-resolved fluorescence spectroscopy. A multi-dimensional fluorescence map that contains spectral, temporal, and intensity information is presented in figure 1*c,d* for both life history stages (gametophyte and tetrasporophyte) after excitation at 400 nm. The fluorescence spectra (figure 1*e,f*), which is given by the emission of the four main algal pigments (chlorophyll, phycoerythrin, phycocyanin and allophycocyanin), obtained by integrating the multi-dimensional map over the whole temporal window (2 ns), exhibited two main peaks, one at 585 nm related to phycoerythrin and the other at 685 nm related to chlorophyll, whereas the contribution from phycocyanin and allophycocyanin appeared as small shoulders for both gametophyte and tetrasporophyte at approximately 640 nm and 660 nm, respectively. electronic supplementary material, figure S1 shows the absorption and emission spectra of the four main pigments. A linear combination of the individual pigment fluorescence spectra was used to fit the measured fluorescence spectra of *C. crispus* from which the relative fluorescence contribution of each pigment was determined (figure 1*e,f*) and the results are tabulated in electronic supplementary material, table S1. The analysis was carried out for both gametophytes and tetrasporophytes, which gave a similar fluorescence composition for both life history phases. Even though it was not possible to assign a specific spectral band to each pigment, due to the partial overlap among the pigments, we identified four spectral ranges where the contribution of each fluorophore was maximized: phycoerythrin (570–590 nm), phycocyanin (630–650 nm), allophycocyanin (650–670 nm), and chlorophyll (680–700 nm).

**Figure 2.**
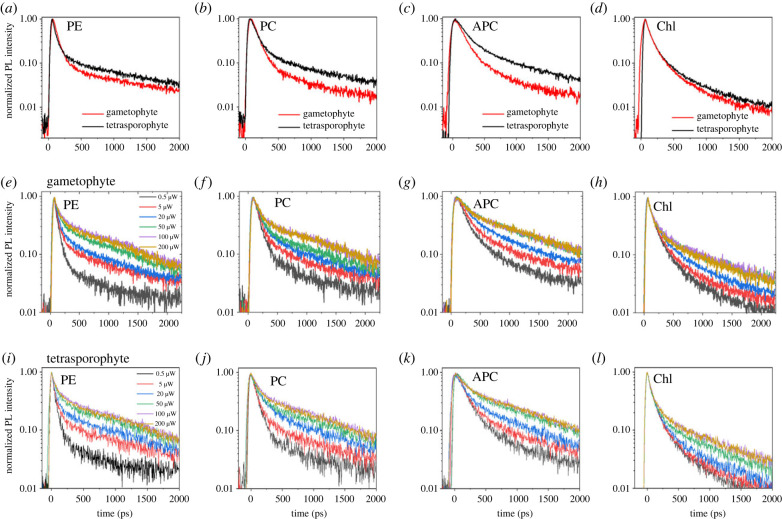
Effect of structural coloration: fluorescence decays for the gametophyte and tetrasporophyte for the different channels exciting at 400 nm and laser power of 5 µW are shown in (*a–d*) and using laser power from 0.5 to 200 µW for gametophytes (*e–h*) and tetrasporophytes in (*i–l*).

The fluorescence temporal decay (electronic supplementary material, figure S2), obtained by integrating the multi-dimensional map over the whole spectral region (550, 720 nm), showed a shorter dynamic in the presence of structural coloration and in particular between 550 and 610 nm, as shown in the multi-dimensional fluorescence map (figure 1*c,d*). The fluorescence temporal profiles of different pigments (figure 2*a–d*) upon excitation at 400 nm (excitation intensity of 5 µW) showed that for phycoerythrin, phycocyanin, and allophycocyanin, the fluorescence decay is faster in the gametophytes than in the tetrasporophytes. For chlorophyll, the fluorescence decay for the gametophytes was slightly faster than for the tetrasporophytes, although the difference between the two life history phases was less pronounced than for the other pigments. As the excitation wavelength was at 400 nm, close to the maximum reflectivity of the gametophytes, the number of photons interacting with the photosystem was different in the presence of structural colour than when absent. Therefore, an intensity-dependent study was performed to investigate the role of excitation intensity on fluorescence kinetics.

The emission spectra, integrated over 0–2 ns, upon excitation at 400 nm with an increase of the laser power from 0.5 to 200 µW, for gametophyte and tetrasporophyte specimens (electronic supplementary material, figure S3*a–b*), showed that with an increase in the excitation intensity, the peak at 660 nm and the shoulder at 640 nm became prominent with respect to the chlorophyll emission. This indicated less energy transfer from the pigments to chlorophyll at higher excitation intensity compared to the lower one. The fluorescence peak at 580 nm, corresponding to the phycoerythrin emission, increased with an increase in excitation intensity which is in agreement with the reported literature for isolated phycobilisomes [34]. To study further the role of excitation intensity, the fluorescence decays for previously selected spectral ranges at varying excitation intensities at 400 nm (figure 2*e–l*), showed that in the case of phycoerythrin, phycocyanin, and allophycocyanin, the increase in excitation intensity caused a slower temporal dynamic up to a maximum above which the intensity dependence vanishes. This observation showed the presence of an intensity-dependent mechanism in the external antennae of *C. crispus*. In the case of chlorophyll, the fluorescence lifetime slightly increased with higher intensities. A similar excitation intensity-dependent fluorescence lifetime for different pigments was observed in the case of gametophytes (figure 2*e–h*) and tetrasporophyte (figure 2*i–l*).

**Figure 3.**
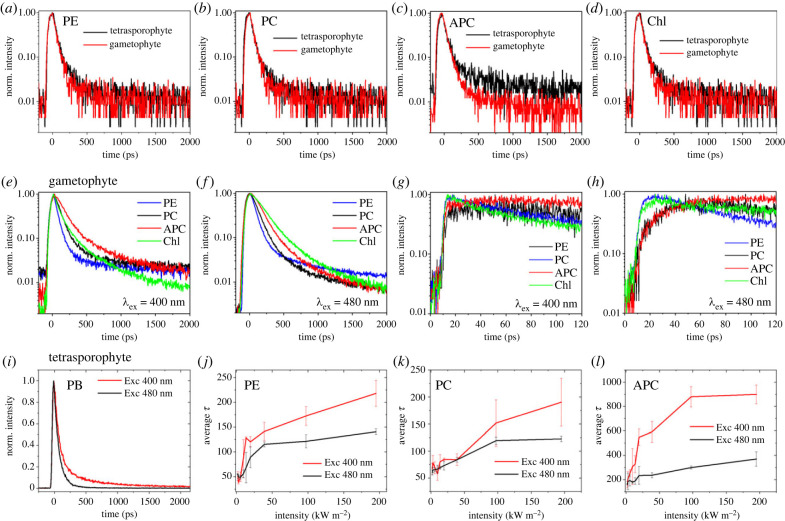
Effect of structural coloration in *Chondrus crispus* gametophytes and tetrasporophytes. Time-resolved fluorescence data of (*a*) phycoerythrin (PE) (*b*) phycocyanin (PC), (*c*) allophycocyanin (APC), and (*d*) chlorophyll (Chl) for gametophytes, with a reflectivity of 0.014, and tetrasporophytes, with a reflectivity of 0.0025, upon excitation at 400 nm with a laser power of 1 µW and 5 µW, respectively. Comparison of the dynamics of gametophytes excited at 400 nm in (*e*) 2 ns and (*g*) 120 ps windows and 480 nm in (*f*) 2 ns and (*h*) 120 ps windows. (*i*) The dynamics of the phycobilisome (PB) band [570-670 nm] upon excitation at 400 nm and 480 nm with a laser power of 5 µW. The mean lifetime (equation (5.3)) and the standard deviation of three replicas of (*j*) PE, (*k*) PC and (*l*) APC are plotted against the excitation intensity at 400 and 480 nm for tetrasporophytes.

The attenuation given by structural coloration in the case of the gametophytes was simulated on the traces of the tetrasporophytes. As the photosynthetic system resides beneath the structural colour multi-layers, which are on the surface, there are fewer photons at around 400 nm that reach the gametophytes than reach the tetrasporophytes. Selecting an illumination intensity for gametophytes and taking into account the reflectivity at around 400 nm for both gametophytes and tetrasporophytes (figure 1*b*), an intensity attenuated by a factor of 5 was selected for the tetrasporophyte to have a similar number of photons reaching the photosynthetic organisms. The fluorescence temporal traces of the different spectral bands upon excitation at 400 nm, with a laser power of 1 µW and 5 µW for tetrasporophytes and gametophytes (figure 3*a–d*), were similar, suggesting that the structural colour has the role of attenuating the photon flux reaching the photosynthetic apparatus of the organism in gametophytes.

To observe the behaviour of the photosynthetic system outside the peak of spectral reflectivity of the structural colour, a wavelength of 480 nm was selected. This allowed the molecular pathways to be investigated when the effect of structural colour was reduced compared to the peak at 400 nm. The spectrum, integrated over time, for excitation at 480 nm, exhibited a stronger peak at 580 nm which was given by phycoerythrin (electronic supplementary material, figure S4), in agreement with the absorption spectrum of the pigments (electronic supplementary material, figure S1). The fluorescence time traces for different spectral regions upon excitation at 400 nm and 480 nm are reported in (figure 3*e,f*). The energy transfer to the reaction centre can follow two pathways: phycoerythrin → phycocyanin → allophycocyanin → chlorophyll or directly from the chlorophyll. As chlorophyll strongly absorbs around 400 nm (Soret band), the second path is predominant in the case of 400 nm excitation compared to 480 nm excitation. The presence of the two pathways was further confirmed by the measurements performed in a shorter time window (120 ps) and higher temporal resolution (approx. 3 ps) which exhibited a build-up in time traces in the case of 480 nm excitation due to energy transfer between the pigments (figure 3*h*), while the build-up cannot be observed in the case of 400 nm excitation (figure 3*g*). A build-up was also present inside the phycoerythrin spectral band that could be attributed to different phycoerythrin proteins [23,29] or from a small contribution of the emission of phycocyanin. A faster decay in the spectral range of the phycobilisome was observed in case of 480 nm excitation compared to 400 nm excitation (figure 3*i*), as with the latter wavelength the chlorophyll was mainly directly excited leading to less energy transfer from the phycobilisomes to chlorophyll and yielding a longer phycobilisome fluorescence lifetime. In order to study the role of 400 nm and 480 nm excitations on the fluorescence lifetimes of individual pigments, the lifetime of different spectral bands representing mainly individual pigments was plotted against varying excitation intensities (figure 3*j–l*).

The fit of the experimental data was made by assuming a global model including a temporal rising component (equation (5.1)) and fixed spectral shapes (equation (5.2)) as described in materials and methods. The results are summarized in electronic supplementary material, table S2 and figures S5*–*S8 for two representative powers, 5 µW and 50 µW. Figure 3*j–l* exhibited an increase in mean lifetime (tau average) for all three pigments upon an increase in excitation power. However, the slope for 400 nm was higher compared to 480 nm excitation revealing a lower energy transfer for all the three pigments by increasing the excitation intensity. electronic supplementary material, figure S9 shows the fitting of five replicas, with associated standard deviation, of gametophyte and tetrasporophyte samples under 400 nm and 480 nm excitations and intensity of 5 µW. It was possible to observe the difference in mean lifetime between gametophytes and tetrasporophytes in the presence of structural coloration (400 nm) for all the PBS pigments. For chlorophyll, a difference was observed but it was not statistically significant. At 480 nm similar mean lifetime for gametophytes and tetrasporophytes was observed. The variations between gametophytes and tetrasporophytes at different intensities exciting at 400 nm is presented in electronic supplementary material, figure S10. Faster dynamics were observed for the gametophyte at all the evaluated powers.

## 3. Discussion

In this study, *in vivo* time-resolved fluorescence measurements on the gametophyte and tetrasporophyte life history phases of *C. crispus* has revealed the role of structural coloration in photoprotection mechanisms in this red alga. In fact, the gametophyte phase shows structural colour with a higher reflectivity around 400 nm as opposed to the tetrasporophyte without structural colour, where the little reflected light that is observed can be due to the scattering from the alga's tissue [48]. These observations are consistent with the study of Chandler *et al.* [11] in their examination of the cuticular structure of *C. crispus* using anatomical and optical approaches.

**Figure 4.**
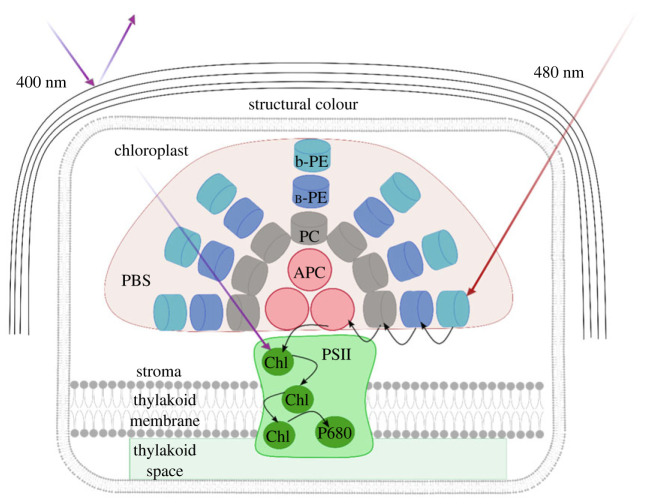
Schematic representation of the antenna systems and principal energy transfer pathways in *Chondrus crispus*. Two main pathways are represented: direct excitation of the chlorophyll with 400 nm excitation (purple arrow) and the indirect excitation of chlorophyll with 480 nm excitation (red arrow). A portion of the light that arrives to the multilayer structure at 400 nm is reflected. The black arrows refer to energy transfer processes.

Faster fluorescence dynamics were observed for the individuals with structural colour compared to those without structural colour when they were excited at 400 nm with the same intensity. In addition, an intensity-dependent mechanism is observed in the external antennae of *C. crispus* for both the gametophyte and tetrasporophyte phase. In detail, upon increase in the excitation intensity, the prominence of the peak at 660 nm and the shoulder at 640 nm with respect to the chlorophyll emission and longer dynamics for the spectral bands related to phycoerythrin, phycocyanin and allophycocyanin pigments for both gametophyte and tetrasporophyte indicate less energy transfer from the pigments to chlorophyll at the higher excitation intensity compared to the lower one. The spectral shapes obtained for *C. crispus* are consistent with those reported for the red algae *Griffithsia pacifica* and *Porphyridium purpureum* [23]. The observation that the same mechanism was observed by exciting outside the wavelength corresponding to the maximum value of reflectivity (at 480 nm) can be attributed to less energy being transferred between the pigments at higher excitation intensity. Previously, Liu *et al.* [34] showed an intensity-dependent phycoerythrin decoupling mechanism in extracted phycobilisomes due to the presence of the *γ* subunit that is sensitive to the intense light and decouples the b-phycoerythrin from the B-phycoerythrin domains of the phycobilisomes [34]. The slight increase in the fluorescence lifetime of chlorophyll can be explained in terms of the higher probability of finding the reaction centre closed at higher intensities [49]. We have observed *in vivo*, an intensity-dependent decoupling for all the three pigments phycoerythrin, phycocyanin, and allophycocyanin, although further investigation is needed to reveal the exact molecular mechanism at the base of the intensity-dependent decoupling of the phycobilisome structure. With intensity reduction, attenuated according to the reflectivity values of structural colour, tetrasporophyte samples (without structural colour) exhibit a similar fluorescence decay to the gametophyte samples. Thus, structural colour acts as an attenuator of the high energy photons. This suggests a possible relationship between structural colour and the intensity-dependent energy transfer mechanism. Under white light illumination, two main pathways are involved in collecting and directing the energy to the reaction centre (figure 4). In pathway (a), the chlorophyll is directly excited, whereas in pathway (b), the pigments of the phycobilisome, once excited, transfer their energy to the chlorophyll. According to the absorption spectra, 400 nm excitation favours path (a) while 480 nm excitation favours path (b). As shown in figure 3*j–l*, at 480 nm excitation, a smaller slope for tau_avg_ *Vs* excitation intensity for phycoerythrin, phycocyanin and allophycocyanin compared to 400 nm excitation is observed. By exciting at 480 nm, the energy transfer (from phycobilisome to chlorophyll) is more probable because the acceptor (chlorophyll) is less saturated compared to the 400 nm excitation.

Therefore, the structural colour and the intensity-dependent mechanism in the phycobilisomes work in synergy to provide an efficient photoprotection mechanism for *C. crispus.* Here, structural colour plays a crucial role in unbalancing the excitation pathways by reducing the energy transfer that arrives at the reaction centre through the direct excitation of the chlorophyll (pathway (a)) and then favouring the relative amount of energy that reaches the reaction centre through the external antennae (pathway (b)).

Red algal light-harvesting complexes are considered to be the ancestor of this protein system in plants and green algae [50]. In particular, the amount of chlorophyll that binds to the protein is smaller than in the green algae [51] and the red alga *Corallina* (now *Ellisolandia*) *elongata* [52] is characterized by the absence of the xanthophyll cycle which is one of the main photoprotective mechanisms in the photosynthetic machinery of the samples*.* Moreover, these organisms lack orange carotenoid protein that is involved in the photoprotective mechanism in cyanobacteria [53,54]*.* In this regard, structural colour can reduce the amount of light that is received directly from this light harvesting complex. The biological significance of regulating the amount of light relates to the protection of the gametophyte stage at higher levels of illumination when a higher concentration of reactive oxygen species (ROS) is produced. ROS can cause oxidative stress which could have a detrimental impact on the nuclei of the sexual reproductive structures in the gametophyte [55]. Further evidence in support of this is that the cells required for sexual reproduction of the organism are located just below the frond tips in the gametophyte which is where the structures responsible for structural colour are found [56].

## 4. Conclusion

By using time-resolved fluorescence spectroscopy *in vivo* to study the role of structural colour in light management in *C. crispus,* we observed the synergistic interaction within different photoprotection mechanisms. Our results indicate that the photonic structure in *C. crispus* works as an attenuator in the gametophyte stage, and that it is able to unbalance the collection of the photon energy, favouring the pathway through the phycobilisomes which can be regulated by an intensity-dependent mechanism. This study provides a better understanding of the mechanism of photosynthetic light harvesting in *C. crispus* and contributes insight into the fine balance of light management within photosynthesis and photoprotection in marine algae.

## 5. Material and methods

### 5.1. Collection of *Chondrus crispus*

Entire individuals of *C. crispus* were collected in the UK from two sites: one tetrasporophyte and five gametophytes from the tidal section of the river Lynher in Antony, Cornwall (50°23′33″ N, 4°13′08″ W) and six tetrasporophytes and six gametophytes from Thurlestone, South Devon, England (50°15′28″ N, 3°51′36″ W). Samples were randomly collected at low spring tide. Tetrasporophytes were identified by the presence of tetraspores on the fronds, while gametophytes were collected when blue structural colour was visible on the tips of the fronds. All the samples were collected and wrapped in wet paper with seawater (on tap at the Marine Biological Association, piped in from Plymouth Sound) for transportation. Then the samples were kept in artificial seawater (made up according to manufacturer's instructions) with 8 h of light and 16 h of dark under at 21°C. The lamp was a NovoLux 60 LED 8 W providing white cool LED (6500 K) light, with an intensity of 15 µmol photons m^−2^ s^−1^. Samples were kept for 20 min in the dark before TRPL measurements.

#### 5.1.1. Time-resolved fluorescence measurements

The time-resolved fluorescence set-up consisted of a light source provided by a Ti : Sapphire oscillator (Chameleon Ultra II, Coherent), producing a train of 140 fs pulses with a repetition rate of 80 MHz. A barium borate crystal (BBO) was used to obtain the second harmonic generation; two absorbing high pass filters (BG39, Thorlabs) were used to remove the residual fundamental excitation. A microscope line was used to achieve suitable spatial resolution. Excitations of 400 and 480 nm were reflected off a suitably chosen dichroic mirror (LP435, LP510, BS50/50) before being coupled into the objective of the microscope and focused onto the sample obtaining an excitation spot diameter of about 30 µm and a mean power of the laser from 0.5 to 200 µW. The light intensities used in this study are presented in electronic supplementary material, table S3. We also note that *Chondrus crispus* is an intertidal species where light levels can be high, particularly in the spring and summer [57], the time of the year when the gametophytes of this alga are undergoing reproduction. *C. crispus* is also influenced by wave action which can create a phenomenon known as the ‘lens effect’, wherein waves act as natural lenses, concentrating and focusing light. As a result, light intensities in this context can be enhanced, ranging from 300% to 500% compared to open-water conditions [58]. Taking this into account, it is reasonable to consider the first two intensity points in electronic supplementary material, table S3 to be below the sunlight intensity, which is standardized sunlight reference at 2000 µmol photons m^−2^ s^−1^ [59]. To gain a more comprehensive understanding of the intensity-dependent processes, we also included measurements under conditions exceeding those of direct sunlight. To achieve high excitation efficiency, a 20 × objective (Zeiss LD EC Epiplan Neofluar, NA 0.22) was used to excite a section of the tip of gametophyte and tetrasporophyte. A section of the tip of each sample of *ca* 1 cm was selected for comparison due to the similarity in pigment composition (electronic supplementary material, table S1). Spectra of the tip, middle and bottom of the same organism were different as shown for a gametophyte specimen in electronic supplementary material, figure S11. Sample emission was collected by the same objective and transmitted through the dichroic mirror, and optical filters (LP455 and LP500) to remove the residual excitation light. The microscope field of view (lateral size of about 120 µm), was selected by a CMOS camera (ORCA-Flash 2.8, Hamamatsu), allowing accurate positioning of the sample relative to the excitation beam via a sample XYZ differential micrometer translation stage. The emission signal was focused on the entrance slit of a spectrograph (Acton SP2300i, Princeton Instrument) coupled to a streak camera (Hamamatsu C5680), resulting in a spectral resolution of around 1 nm and temporal resolution of 20 ps (for 2 ns time window).

The sample number for TRPL measurements (spectra and temporal dynamics) is as follows: for 400 nm excitation and 5 µW, the number of measurements were 23 for gametophyte tips (two tips each from 10 samples and three tips from one sample; in total 11 gametophytes samples) and 23 for tetrasporophyte tips (three tips each from five sample and four tips each from two samples; in total seven tetrasporophytes); more than one tip were taken from a specimen in order to ensure a large enough sample numbers. For 480 nm excitation and 5 µW, five replicas of gametophyte (five tips from five gametophyte samples) and five replicas of tetrasporophyte (five tips from five tetrasporophyte samples). For the intensity dependent at 400 nm and 480 nm, three replicas (three tips from three different samples) at each intensity for both gametophyte and tetrasporophyte.

The experimental temporal traces of each spectral band were fitted using equation (5.1) for all the pigments (for both excitation at 400 and 480 nm). The fitting equation shows a rising component to model the energy transfer process.

5.1 $$f(t)=(A_{1}e^{-t/\tau_{1}}+A_{2}e^{-t/\tau_{2}})\times (1-\alpha e^{-t/\tau_{3}}).$$

The theoretical curves were convoluted with a Gaussian instrument response function (IRF) with a full width at the half maximum of 20 ps. The spectral curves integrated over the time (2 ns window) were fitted by a linear combination of the fluorescence spectra of each pigment. The results of the independent temporal and spectral fitting were used as starting parameter for a global fitting of the process by using the model described by equation (5.2).

5.2 $$\varphi (t,\lambda )=\sum_{i=1}^{4}c_{i}(t)\;*\;\varepsilon_{i}(\lambda ),$$

where the spectral shape ($\varepsilon_{i}(\lambda )$) of each spectrum was fixed to the experimental values reported in the literature and only the amplitudes are fitted. The temporal shape $c_{i}(t)$ is reported in equation (5.1).

For comparing the temporal dynamics of each pigment, the mean lifetime *τ* was calculated (equation (5.3)) by considering the decay *τ*_1_ and *τ*_2_.

5.3 $$\mathrm{Average}\;\tau =\frac{\left(A_{1}\times \tau_{1}+A_{2}\times \tau_{2}\right)}{\left(A_{1}+A_{2}\right)}.$$

#### 5.1.2. Reflectivity measurements

Optical imaging and microspectrometry were performed using a customized Zeiss Axio Scope A1 equipped with a 40× water immersion objective (Zeiss,W N-Achroplan, NA 0.75) and using a halogen lamp as the light source. Images were acquired with a CMOS camera (Pixelink, PL-D725CU-T, calibrated against a white standard) and reflectance spectra were recorded by coupling the microscope to a spectrometer (Avantes, AvaSpec-HS2048) with an optical fibre (Avantes, FC-UVIR200-2-SR, 200 μm core size). Reflectance spectra were taken using a spectralon (AS-01159-060) and normalized against a silver mirror (Thorlabs, PF10-03-P01). Five different micrographs and reflectance spectra were collected for each sample, and seven samples of gametophytes and seven samples of tetrasporophytes were observed.

The reflectivity was monitored during the photoluminescence measurements with a similar set-up. A UV-Vis fibre light source (Hamamatsu, L10290) was coupled into the microscope line of the time-resolved photoluminescence set-up using a 50/50 beam splitter and a 20× objective (Zeiss LD EC Epiplan Neofluar, NA 0.22). Reflectivity spectra were collected using a high-sensitivity spectrometer (Maya 2000 Pro) and normalized against a silver mirror (Thorlabs, BB1-E02-10). The number of replicas for reflectivity measurements is 35 gametophyte tips from 11 samples and 35 tetrasporophyte tips from seven samples.

Time-resolved photoluminescence measurements and the corresponding reflectivity spectra were recorded for 23 samples of gametophyte and 23 samples of tetrasporophyte.

## Data Availability

The data that support the findings of this study are available within the article and the electronic supplementary material [60].
